# Multisite Comparison of MRI Defacing Software Across Multiple Cohorts

**DOI:** 10.3389/fpsyt.2021.617997

**Published:** 2021-02-24

**Authors:** Athena E. Theyers, Mojdeh Zamyadi, Mark O'Reilly, Robert Bartha, Sean Symons, Glenda M. MacQueen, Stefanie Hassel, Jason P. Lerch, Evdokia Anagnostou, Raymond W. Lam, Benicio N. Frey, Roumen Milev, Daniel J. Müller, Sidney H. Kennedy, Christopher J. M. Scott, Stephen C. Strother, Stephen R. Arnott

**Affiliations:** ^1^Rotman Research Institute, Baycrest Health Sciences Centre, Toronto, ON, Canada; ^2^Ontario Brain Institute, Toronto, ON, Canada; ^3^Department of Medical Biophysics, Robarts Research Institute, Western University, London, ON, Canada; ^4^Department of Medical Imaging, Sunnybrook Health Sciences Centre, Toronto, ON, Canada; ^5^Department of Psychiatry, Cumming School of Medicine, University of Calgary, Calgary, AB, Canada; ^6^Mouse Imaging Centre, Hospital for Sick Children, Toronto, ON, Canada; ^7^Bloorview Research Institute, Holland Bloorview Kids Rehabilitation Hospital, Toronto, ON, Canada; ^8^Department of Psychiatry, University of British Columbia, Vancouver, BC, Canada; ^9^Department of Psychiatry and Behavioural Neurosciences, McMaster University, Hamilton, ON, Canada; ^10^Mood Disorders Program, St. Joseph's Healthcare, Hamilton, ON, Canada; ^11^Departments of Psychiatry and Psychology, Queen's University, Providence Care Hospital, Kingston, ON, Canada; ^12^Molecular Brain Science, Campbell Family Mental Health Research Institute, Centre for Addiction and Mental Health, Toronto, ON, Canada; ^13^Department of Psychiatry, University of Toronto, Toronto, ON, Canada; ^14^Department of Psychiatry, Krembil Research Centre, University Health Network, Toronto, ON, Canada; ^15^Department of Psychiatry, St. Michael's Hospital, University of Toronto, Toronto, ON, Canada; ^16^Keenan Research Centre for Biomedical Science, Li Ka Shing Knowledge Institute, St. Michael's Hospital, Toronto, ON, Canada; ^17^LC Campbell Cognitive Neurology Research Unit, Toronto, ON, Canada; ^18^Heart & Stroke Foundation Centre for Stroke Recovery, Toronto, ON, Canada; ^19^Sunnybrook Health Sciences Centre, Brain Sciences Research Program, Sunnybrook Research Institute, Toronto, ON, Canada; ^20^Department of Medical Biophysics, University of Toronto, Toronto, ON, Canada

**Keywords:** de-identification, structural MRI, facial recognition, 3D rendering, defacing, privacy—preserving

## Abstract

With improvements to both scan quality and facial recognition software, there is an increased risk of participants being identified by a 3D render of their structural neuroimaging scans, even when all other personal information has been removed. To prevent this, facial features should be removed before data are shared or openly released, but while there are several publicly available software algorithms to do this, there has been no comprehensive review of their accuracy within the general population. To address this, we tested multiple algorithms on 300 scans from three neuroscience research projects, funded in part by the Ontario Brain Institute, to cover a wide range of ages (3–85 years) and multiple patient cohorts. While skull stripping is more thorough at removing identifiable features, we focused mainly on defacing software, as skull stripping also removes potentially useful information, which may be required for future analyses. We tested six publicly available algorithms (afni_refacer, deepdefacer, mri_deface, mridefacer, pydeface, quickshear), with one skull stripper (FreeSurfer) included for comparison. Accuracy was measured through a pass/fail system with two criteria; one, that all facial features had been removed and two, that no brain tissue was removed in the process. A subset of defaced scans were also run through several preprocessing pipelines to ensure that none of the algorithms would alter the resulting outputs. We found that the success rates varied strongly between defacers, with afni_refacer (89%) and pydeface (83%) having the highest rates, overall. In both cases, the primary source of failure came from a single dataset that the defacer appeared to struggle with - the youngest cohort (3–20 years) for afni_refacer and the oldest (44–85 years) for pydeface, demonstrating that defacer performance not only depends on the data provided, but that this effect varies between algorithms. While there were some very minor differences between the preprocessing results for defaced and original scans, none of these were significant and were within the range of variation between using different NIfTI converters, or using raw DICOM files.

## Introduction

With the rising prominence of data sharing and large-scale medical studies, proper removal of protected health information (PHI) is paramount to preserving the privacy of study participants. Text identifiers, such as participants' names, date of birth, sex, etc. are already commonly removed, but one growing concern is the ability to recognize a person, based on their face, as rendered from a structural magnetic resonance image (MRI) (1, 2), arguably falling within the Health Insurance Portability and Accountability Act (HIPAA) requirement of removing “full-face photographs and any comparable images” from collected data to be considered de-identified (3). In a 2019 experiment conducted by the Mayo Clinic, facial recognition software correctly matched 3D renders from 83% of participant scans to their corresponding photographs (1). While it can be argued that this experiment may not be wholly representative of standard concerns for data breaches, as in this set-up there was the artificial foreknowledge that the scans must belong to one of 84 participants, this is still a worryingly accurate rate. Combining the constant push for higher spatial resolution and quality of MRI scans, with improvements in facial recognition software, such accuracy is only expected to increase over the coming years.

Unlike text identifiers, which can simply be removed, replaced by generic codes or randomly generated IDs, or blurred with ranges as in the case of numeric values such as dates or ages, removal of faces is more complicated. Voxels containing data which could be used to reconstruct recognizable features must be removed, yet regions of interest must also remain intact. Depending on the research goals for the collected scans, these regions of interest may also change. Skull stripping is a common and thorough method for handling this, with many available methods to choose from (summary in Table 1), however, for certain studies, the skull or other non-neuronal tissues are essential for preprocessing or for particular analyses. One example of this, are the landmarks within the skull and along the scalp that are used in combined MRI and EEG/MEG studies to align the multi-modal data (11, 12). With respect to direct analyses, measurements, such as total cerebral spinal fluid (CSF) and total intracranial volume, also require tissue that skull stripping inherently removes (13). While some of these values may be collected before the skull-stripping is completed and provided along with the scans themselves, for this to occur there must be advanced knowledge of which factors would be required, limiting future use of the data. Accurate comparison of these pre-calculated values across multiple datasets may also be impossible, if different skull stripping software have been used, as there is a noticeable difference between measurements made by various methods (14), especially when handling patient data (15). For these instances, removing only the facial features, i.e., defacing, may be a more suitable approach (13), as it leaves the rest of the scan intact and is a method currently adapted by several large-scale neuroimaging projects (16, 17).

**Table 1:** Summary of several commonly used skull-stripping algorithms for T1-weighted images.

**Algorithm**	**Description of method**
Brain extraction tool (BET) (4)	Deformable model which expands from an estimated center of gravity until the brain surface is reached, based on intensity-driven estimates of brain vs. non-brain thresholds. Fractional intensity threshold, its vertical gradient, head radius and center of gravity can be adjusted by the user to improve results.
RObust, learning-based Brain EXtraction (ROBEX) (5)	Learning model using combined generative and discriminative models. Fully data-driven; no user-supplied parameters.
AFNI 3dSkullStrip (6)	Modified version of BET, using non-uniformity correction and edge detection to reduce errors. Provides multiple parameters and flags that the user can adjust to improve skull strip.
Brain surface extraction (BSE) (7)	Uses Marr–Hildreth edge detection after anisotropic diffusion filtering to improve boundary contrast. Semi-automated—displays intermediate results to allow for parameter tuning of filter and edge detector
antsBrainExtraction (8) (https://github.com/ANTsX/ANTs)	Completes brain extraction using N4 intensity normalization, a template and probability map. User must determine which template and brain probability maps work best for their data, although sample files are provided on download site.
FreeSurfer (9)	Combination of watershed (intensity based), deformation and atlas-based techniques to identify and extract brain tissue. User can adjust seed point and watershed threshold, if required.

*For a more detailed and comprehensive list of skull-stripping techniques, refer to the following review by Kalavathi and Surya Prasath (10)*.

Another existing method is facial blurring, where voxels that are identified as containing facial features are blurred, rather than removed. This method preserves the most information and by using morphological features rather than registering a mask to remove the subject's face, this method also reduces the risk of removing or altering brain tissue (18, 19). While this might sufficiently distort features so that visual recognition is not possible from straight 3D renders of the blurred scans (18), with the right models it is possible to reverse this blurring and recreate the original scan (20), rendering such methods useless in preserving patient privacy. As such, all de-identification algorithms that relied on this method, were excluded from this study.

The final method, defacing, is similar to facial blurring, except that voxels containing facial features are removed, rather than blurred, eliminating the possibility of reversing the deidentification (20). There have been numerous, publicly available software algorithms that have been developed using this method (13, 21–23) (descriptions in Table 2), and while there have been a few reports on the success of individual defacers (13, 24), there has not been a systematic review of the available choices and how they perform across scans in different populations. In this study, we sought to fill that gap, by examining the performance of different defacing algorithms across a wide range of structural scans. These results will be useful to inform consortia, such as Ontario Brain Institute (OBI)'s Brain Centre for Ontario Data Exploration [i.e., Brain-CODE (25–27)], on the best approaches for maintaining participant privacy within publicly shared datasets.

**Table 2:** Summary of the method used for each algorithm to deface scans, with an example sagittal slice after defacing has been applied.

**Defacer**	**Method for defacing**	**Example slice**
afni_refacer	Pre-defined mask aligned using AFNI 3dAllineate and MNI template (6)	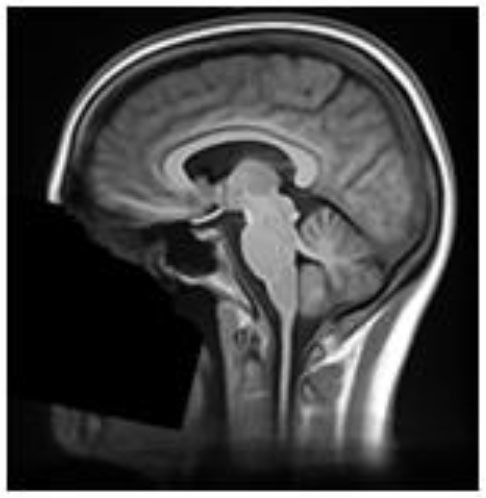
deepdefacer	Pre-defined model of facial probabilities used to calculate probabilities of facial features within a region. This is used to create a binary mask to remove facial features (22)	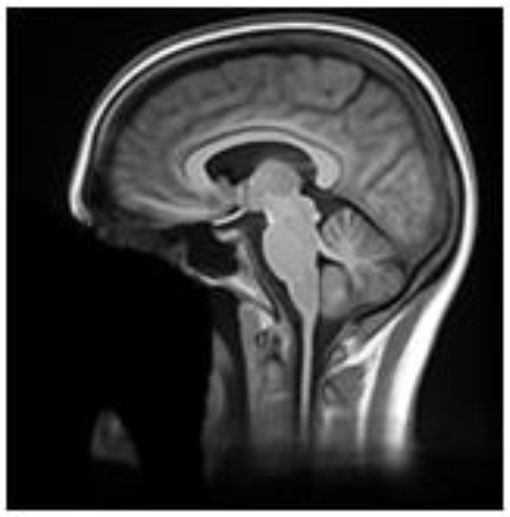
mri_deface	Assigns probability of voxel being “face” or “brain” and removes voxels that have non-zero probability of being “face” but zero probability of being “brain” (13)	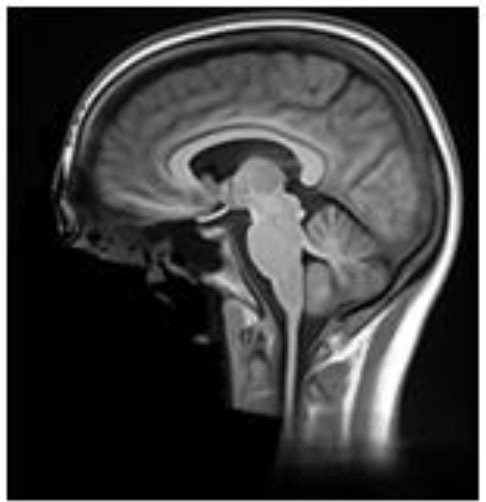
mridefacer	Skull strips input scan, and aligns result with pre-defined mask using FSL FLIRT and a template T1 brain, then applies mask to original scan to remove “face” and “ear” voxels (https://github.com/mih/mridefacer)	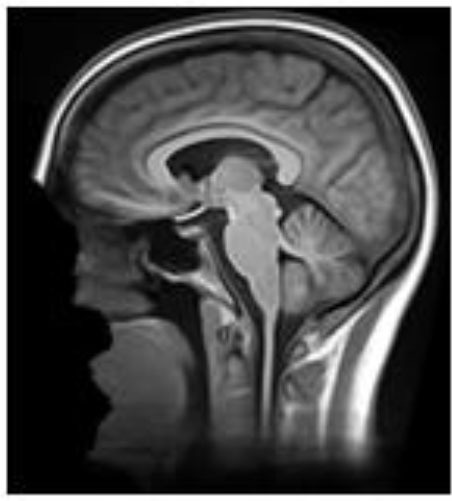
pydeface	Aligns pre-defined mask, using FSL FLIRT and a template T1 structural scan, to the input scan and removes “face” voxels (23)	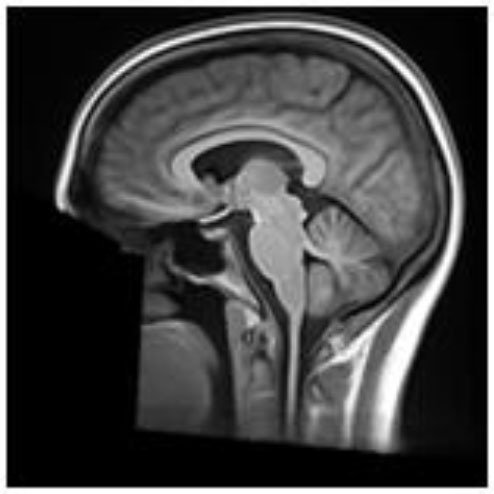
quickshear	Uses previously created brain mask to draw a plane between “face” and “brain” and removes all voxels on the “face” side. A “buffer” parameter is used to set the number of voxels between the plane and the edge of the brain mask, (default:10) (21). All scans in this study were defaced using this default.	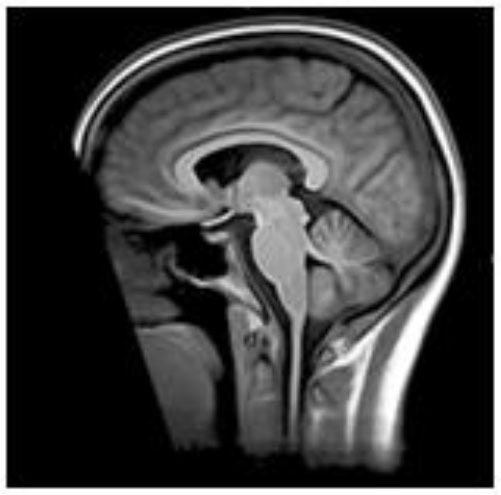

## Materials and Methods

### Measuring Defacer Success

One hundred T1-weighted structural MRI scans were randomly selected from each of three of OBI's multisite datasets (25), for a total of three hundred scans, chosen to span different age groups and patient cohorts—the Ontario Neurodegenerative Disease Research Initiative (ONDRI) (25, 28), the Canadian Biomarker Integration Network in Depression (CAN-BIND) (29, 30) and the Province of Ontario Neurodevelopmental Disorders Network (POND) (31, 32) [scan parameters previously described in (28, 32, 33), demographic details in Table 3]. Incomplete scans, as well as those with severe motion or imaging artifacts, were excluded prior to selection, as those scans would inevitably be excluded from future analyses and could potentially skew success rates for datasets (e.g., incomplete scans marked as having brain removed, which the algorithm would normally have left intact, or scans marked as defaced because motion or imaging artifacts obscured remaining facial features). Each scan was then run through six different publicly available defacing programs [@afni_refacer_run v2.2 (6), deepdefacer v2.1.2 (22), pydeface v2.0.0 (23), mri_deface v1.22 (13), mridefacer v0.2 (https://github.com/mih/mridefacer), quickshear v1.1.0 (21), descriptions in Table 2] and one skull stripper, FreeSurfer v6.0 (9), for comparison. Defaced scans were then manually reviewed in Mango (34) by three independent raters, to ensure that the algorithm had not removed any brain tissue. Viewer3D in MATLAB R2016b (35) was used to generate 3D rendered images (5 per scan—straight on, and at 30° and 45°, left and right) to determine whether or not a recognizable face remained. Defacing was considered to be successful if (1) the 3D render did not contain more than one partial facial feature (eyes, nose, or mouth) and (2) no brain tissue had been removed during defacing. Success rates were then compared between defacing software and each of the datasets. Inter-rater reliability was measured using percent agreement and free-marginal kappa (36, 37).

**Table 3:** Participant demographics of scans used in testing defacer accuracy.

**Dataset**	**Age range, mean**	**Diagnosis groups**	**# of scanners**
POND	3–20, 12.1 ± 3.7 years	71 Autism Spectrum Disorder, 11 Attention Deficit Hyperactivity Disorder, 5 Obsessive Compulsive Disorder, 13 Healthy Control	2 Siemens
CANBIND	18–60, 34.9 ± 12.8 years	44 Major Depressive Disorder, 56 Healthy Control	4 GE, 1 Siemens, 1 Philips
ONDRI	44–85, 69.6 ± 8.4 years	33 Alzheimer's Dementia or Mild Cognitive Impairment, 11 Amyotrophic Lateral Sclerosis, 13 Frontotemporal Dementia, 17 Parkinson's Disease, 26 Stroke	3 GE, 6 Siemens, 1 Philips

*One of the Siemens scanners was used in data collection for all three datasets, while two of the GE scanners were the same for CANBIND and ONDRI. All scanners were 3T models*.

The initial defacing threshold was set at no facial features remaining within the 3D render, but was later relaxed to no more than a single partial feature, due to lack of recognizability within render and poor rater agreement over what qualified as “fully defaced” vs. “single facial feature remaining.” Original results can be found in Supplementary Figure 1 and Supplementary Table 1.

Because we did not have any photographs of these participants to test automated facial recognition with (1, 2), we instead used facial detection within the generated 3D renders, as an estimate of whether or not a scan still contained features a computer could use to identify the participant after defacing. The deep neural networks (DNN) module for the OpenCV v4.1.2 package, with the default pre-trained face detection model, res10_300x300_ssd_iter_140000.caffemodel (38), in Python v3.6.4 (39) was used to generate a confidence level that there was a face within the 3D render. These were then compared between the defacers, as well as with the levels generated for the renders of the original, pre-defaced scans.

### Testing Facial Recognition

To examine true facial recognition, nine human raters were asked to complete an online (Google Forms) 3D render MRI recognition task. Since we were unable to collect photographs for the participants in the previous 300 scans, another, more recently-collected dataset was leveraged. Structural MRI scans using the ONDRI 3DT1 protocol (40) [scan parameters same as (28)] were obtained from six participants (ages: 46–64, mean 56.5 years old) who participated in the OBI's Traveling Human Subject Study (THSS) and who gave consent to have their photographs and MRI renders to be used for this purpose. Three of these participants were personally familiar to the nine raters (mean familiarity 3.9 ± 5.7 years), while the remaining three participants were not familiar to the raters. Each participant had undergone scans from the same 12 OBI-affiliated 3T MRI scanners, for a total of 68 3DT1 scans (note, two subjects only completed scans at 11 sites and two additional scans were omitted from the final quiz).

**Figure 1:**
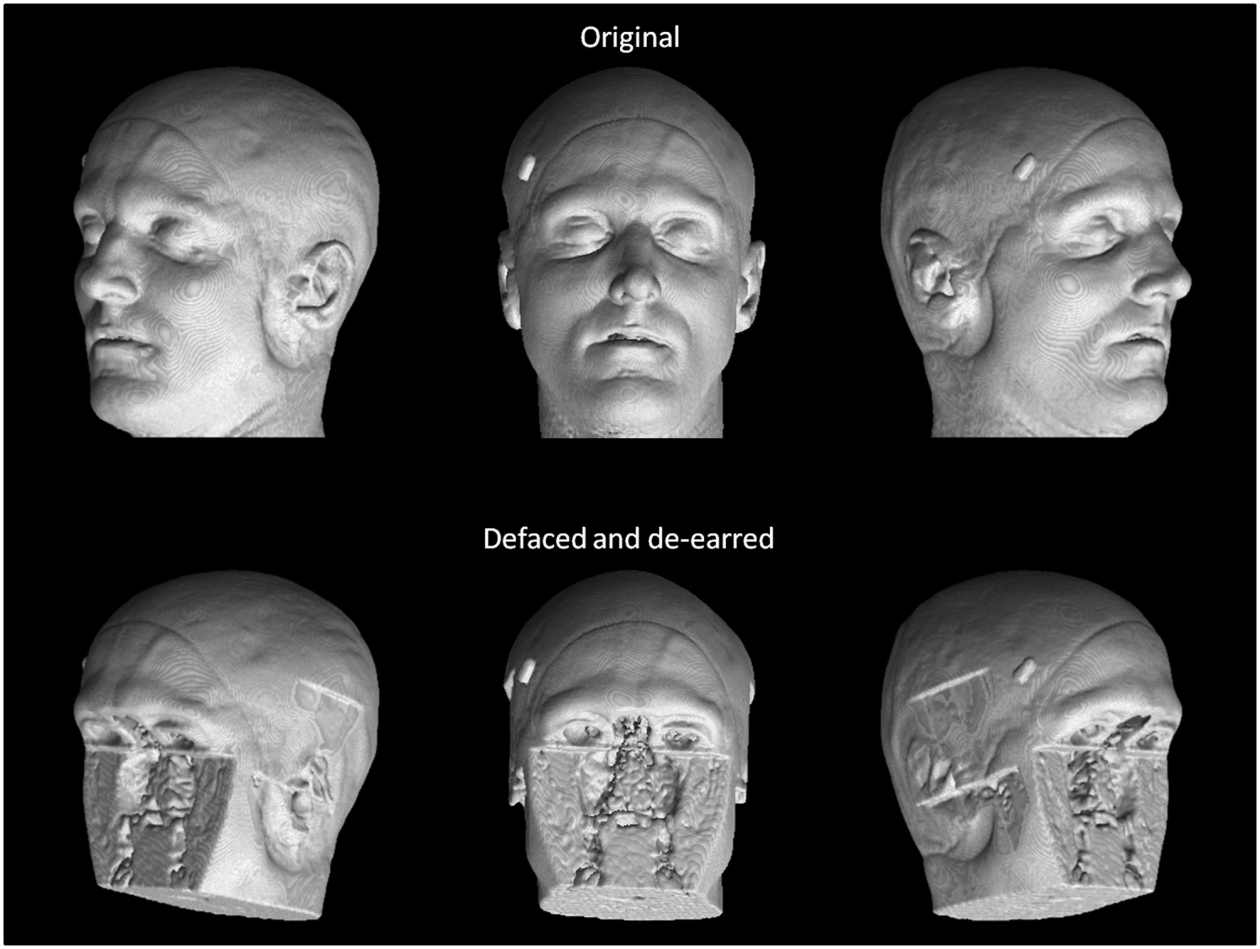
True facial recognition task images. Top row: Sample original (pre-defaced) 3D rendered T1 image. Three perspectives of the head were generated, including 45° left, straight on, and 45° right. Bottom: The same image after undergoing defacing (in this case, pydeface) and de-earring. Consent was given by the participant to include their non-defaced MRI render in the publication.

These 68 scans underwent defacing using each of the six defacing algorithms outlined in section Measuring Defacer Success. Following the defacing procedure, each image underwent an additional de-earring step using ear masks generated with fsl_deface (16). For each of the defacing sets, three of the twelve scans from each participant were randomly chosen for the recognition task, for a total test set of 108 defaced images. Two participants were used as “unknowns,” while photographs of the remaining four participants (including the three participants already personally familiar to the raters) were provided to human raters who then attempted to identify the 108 randomly presented defaced images. To help with recognition, each image contained three perspectives of the same 3D render (45° left, straight on, and 45° right of where the face would be; see Figure 1). For each image, raters were instructed to select one of six responses indicating whether they recognized the image as belonging to the person pictured in photograph 1, photograph 2, photograph 3, photograph 4, none of the four photographs, or whether there was not enough information available to make a confident recognition judgment (i.e., “Can't identify”).

Once raters had completed the defaced scan recognition questions, they were then allowed a break before they began the recognition task of the original pre-defaced scans. Defaced image identification always occurred prior to original image identification so as to avoid the possibility of any learned associations being gleaned from non-defaced images (e.g., skull features or markings unique to an individual). Following the recognition ratings, raters provided answers to debriefing questions asking how difficult they found the task, what cues and strategies they had employed during recognition, as well as how personally familiar each of the four persons in the photographs were to them.

Automated facial recognition was attempted using Microsoft Azure (https://azure.microsoft.com/en-us/services/cognitive-services/face/), similar to the procedure of a previous study (1), however, either due to scan quality or distortion of participants' heads within the coil, this software was unable to locate faces within our renders, even for those which had not yet been defaced, making it impossible to compare them to actual photographs. In future, other methods of automated facial recognition may be explored, but for this study, only the manual ratings were used.

### Testing Effects on Preprocessing Pipelines

One concern with defacing images, beyond direct errors made by the algorithms itself, is that the use of defaced MRI in preprocessing pipelines and analyses may alter the results (19). Depending on how the data are processed, the missing facial features could introduce variations to the output that might skew subsequent analyses, especially when trying to compare or pool two datasets where one had been previously defaced and one had not. To address this, several preprocessing pipelines were explored using a subset of the THSS sessions. Defaced T1s were processed through each pipeline following the same steps and parameters as for the original scans, and the results compared to see if there were any significant variations. To provide a baseline for this comparison, the raw DICOM files of the pre-defaced image and the NIfTI file created using a different converter—dcm2niix (41) vs. Python's dicom2nifti (42)—were also run through the same pipelines and similarly compared to the original input.

#### FreeSurfer

Effects on T1 tissue segmentation and signal normalization were examined using FreeSurfer's recon-all (9). Total brain, intracranial, cortical and subcortical gray matter, and white matter volumes were extracted for each case, as well as average left and right hemisphere cortical thickness, and cortical gray-to-white matter contrast-to-noise ratio (CNR), defined as:


$$\begin{array}{l} CNR=\frac{{\left(WM_{Avg}\;-\;GM_{Avg}\right)}^{2}}{\left(WM_{Var}\;+\;GM_{Var}\right)} \end{array}$$

where GM and WM are the cortical gray and white matter signal intensities, as demarcated by FreeSurfer's aseg file. Additionally, the percent overlap between the brain masks for each of the defaced and alternate file formats, and the original scan, were calculated for segmented cortical and subcortical gray matter and white matter tissue, as defined by the following equation.


$$\begin{array}{l} \%\;Overlap=\frac{\left(T_{D}\;\cap \;T_{O}\right)}{\left(T_{D}\;\cup \;T_{O}\right)} \end{array}$$

Where T_O_ is the segmented tissue for the original brain mask and T_D_ is the segmented tissue for the brain mask of the scan being compared.

#### fMRI Preprocessing

Functional MRI (fMRI) (scan parameters in Table 4) and T1 scans for 19 sessions were processed through the Optimization of Preprocessing Pipelines for NeuroImaging (OPPNI) (43, 44), which uses the structural scans to register functional scans to a common space. Resultant statistical parametric mapping (SPM) files were then compared using FSL Randomize with family-wise error correction (45) to see if using defaced T1s for registration made any significant difference to results.

**Table 4:** MRI scan parameters for fMRI scans.

**# of scans**	**Scanner**	**TR (ms)**	**TE (ms)**	**FOV, slices**	**Resolution (mm)**	**α (^**°**^)**
19	TrioTrim	2,400	30	448x448,250	3.5x3.5x3.5	70

*TR, repetition time; TE, echo time; FOV, field of view; α, flip angle*.

#### Image Registration

The final preprocessing aspect examined was the direct registration of images to a common space. To do this, the brain masks previously generated through FreeSurfer were aligned to the MNI152 2 mm template using FLIRT (46) with 12° of freedom (affine). Since all of the defaced scans should start in the same position as the original, the 12 alignment parameters were then extracted to see how accurately the scans would match after registration. For the DICOM and dcm2niix brain masks, the translation parameters were adjusted to match the 1 mm offsets of their centers' locations compared to that of the original brain mask, before comparison.

All plots were generated using Seaborn v 0.9.0 (47) in Python v3.6.4 (39) or ggplot2 v3.2.1 (48) in R v3.6.3 (49). Statistical analyses for FreeSurfer and FLIRT outputs were conducted in R.

## Results

### Manual Ratings

**Figure 2:**
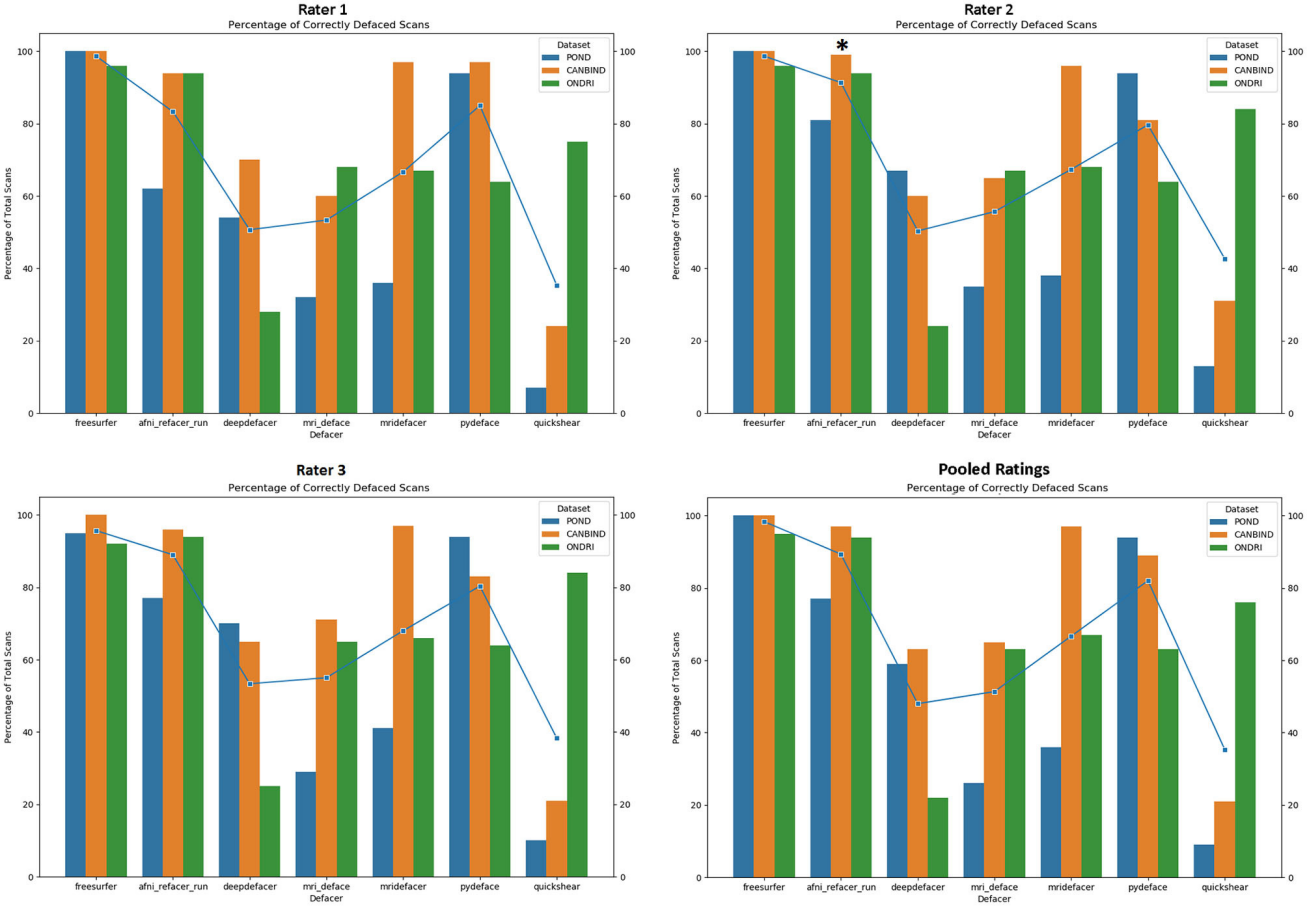
Percentage of scans passed by each rater, split by dataset and defacing algorithm. Markers indicate the average percentage for each algorithm. Pooled ratings indicate the percentage of scans that passed based on rater consensus for each scan. *Disclaimer: afni_refacer_run ratings had to be redone due to a major software update after initial data collection. Due to the unavailability of the original Rater 2, these ratings were completed by a different person.

As expected, FreeSurfer had the highest accuracy for successfully removing facial features (98.7%), with the sole source of error originating from brain clipping within the ONDRI cohort (Figure 2). Focusing on the defacer software, afni_refacer and pydeface performed the best on average (89 and 83% respectively), although performance seemed to drop with the POND cohort for afni_refacer (77%), while pydeface's performance seemed to suffer with ONDRI (64%). Of all the algorithms, quickshear performed the worst, with an average pass rate of only 39% due to its frequent failure to remove eyes, and sometimes even mouths. Although these factors were not used to determine pass or failure, quickshear also had a tendency to leave other possibly identifiable facial structures such as cheeks and jawline, especially within the younger cohorts. Examples of successfully, and incorrectly, defaced scans can be seen in Supplementary Table 2.

**Figure 3:**
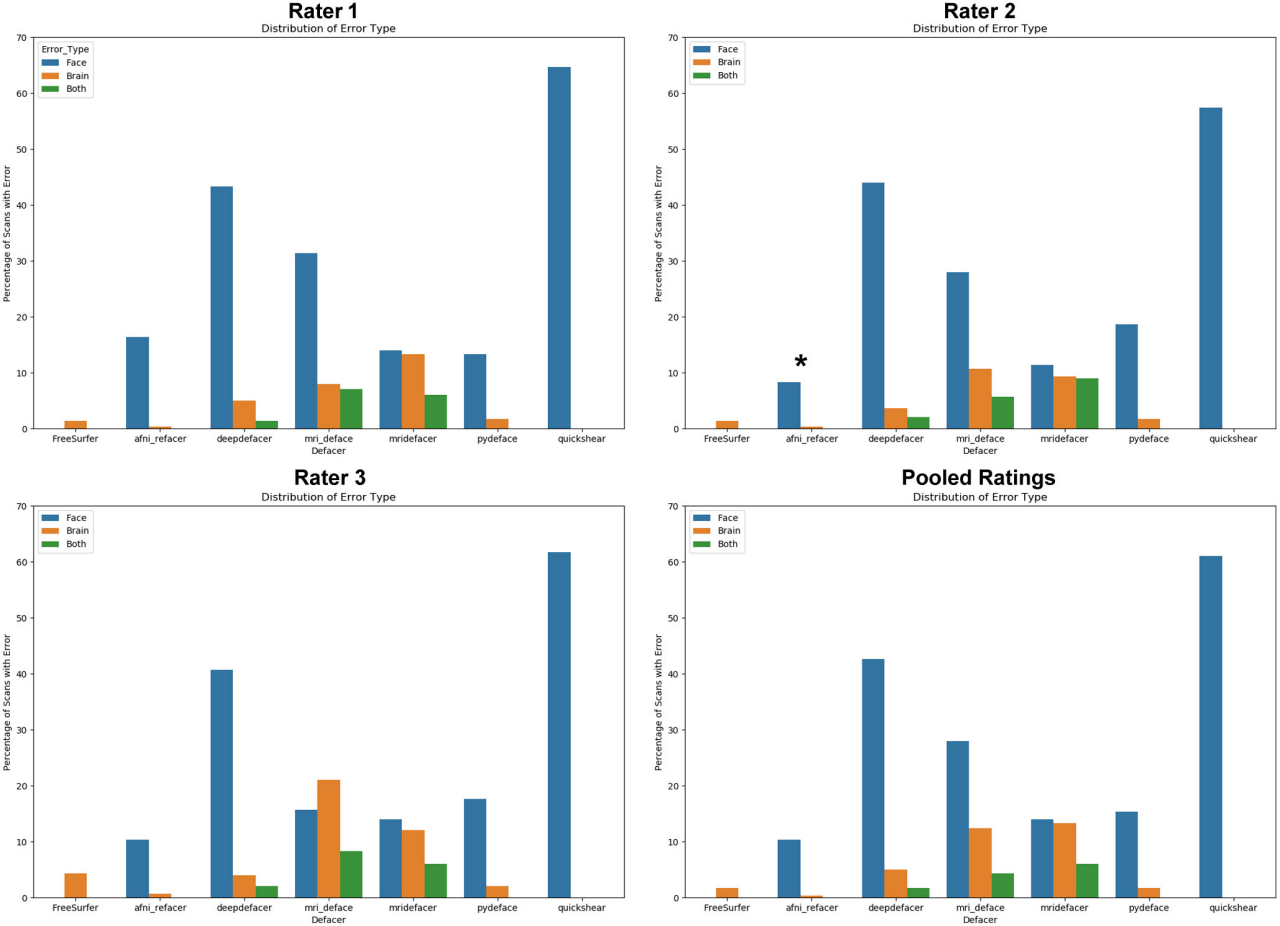
Percentage of scans where errors were detected for each of the seven algorithms, split based on error class. “Face” refers to scans that were failed due to at least one identifiable facial feature (eyes, nose, mouth) remaining after defacing, “brain” refers to scans that were failed due to the algorithm removing neuronal tissue, while “both” references scans where both of these errors occurred. Pooled ratings were calculated from the rater consensus for each scan. *Disclaimer: afni_refacer_run ratings had to be redone due to a major software update after initial data collection. Due to the unavailability of the original Rater 2, these ratings were completed by a different person.

The most frequent source of error was missed facial features (Figure 3), with only FreeSurfer (i.e., skull stripping) failing solely due to brain removal, and mridefacer with an almost even split between the missed facial features (51%) and brain removal. In all other cases, brain removal by the algorithms was rare, accounting for only a little more than 10% of the remaining errors. When brain removal did occur, the amount was usually fairly low, averaging at around 0.47 ± 0.9% of brain voxels removed for most algorithms and primarily occurred around the frontal pole and along the lateral surface of the temporal lobe. The one exception was mridefacer, which frequently removed a much higher amount, averaging at 6.3 ± 17% of brain voxels removed, with one scan going as high as 78%, due to an alignment failure.

#### Inter-rater Reliability

Agreement was fairly high between raters for most defacers (Table 5); however, this agreement dropped for mri_deface, probably due to the shape of the mask. While most of the defacers created a smooth boundary where they removed voxels, mri_deface created a very jagged edged, at times even disjointed, mask (Table 2), which added a two-fold difficulty in rating. For one, when it removed brain voxels, the amount removed tended to be very small, which could be missed by some of the raters. The other confound was that when it failed to remove facial features, it typically still removed part of the feature, which led to disagreement between raters on whether or not this counted as identifiable. As such, this particular defacer was more difficult to rate, leading to the higher discrepancy between raters.

**Table 5:** Inter-rater reliability for manual ratings of each dataset and algorithm, as measured using percent agreement and free-marginal kappa.

**Defacer**	**Percent agreement**			**Free marginal kappa**		
	**POND**	**CANBIND**	**ONDRI**	**POND**	**CANBIND**	**ONDRI**
FreeSurfer	96.7	100	94.0	0.933	1.00	0.880
afni_refacer	88.7	96.0	100	0.747	0.920	1.00
deepdefacer	84.0	86.0	88.7	0.680	0.720	0.773
mri_deface	73.3	80.7	82.7	0.467	0.613	0.653
mridefacer	92.7	99.3	97.3	0.853	0.987	0.947
pydeface	98.7	84.0	97.3	0.973	0.680	0.947
quickshear	95.3	86.0	92.0	0.907	0.720	0.840

### Automated Face Detection

**Figure 4:**
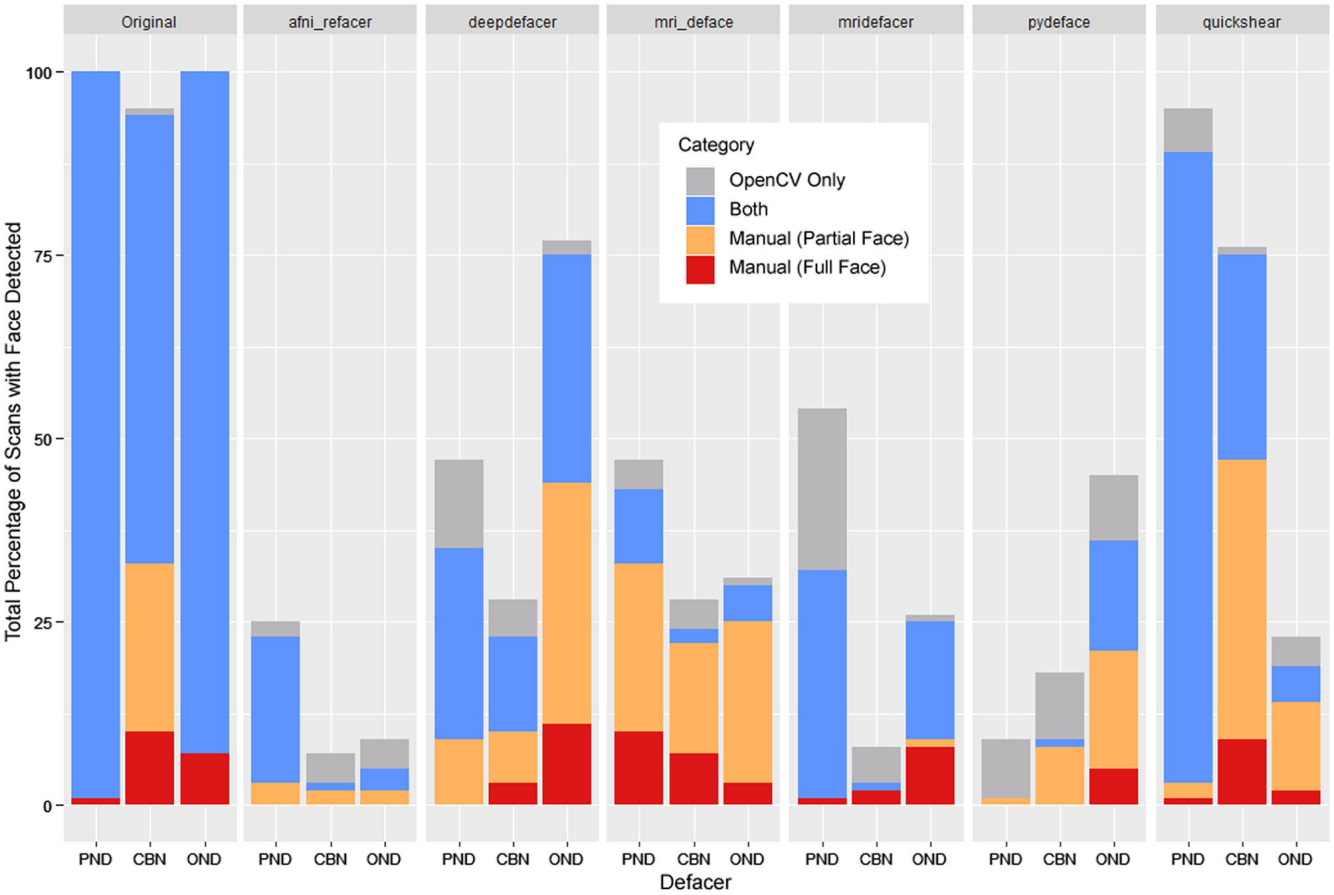
Total percentage of scans where OpenCV or human raters detected a face within the 3D render, segmented by whether a face was detected by both (blue), OpenCV only (gray), or through manual ratings only—subdivided into partial faces (1 feature—orange) and full faces (2+ features—red). FreeSurfer was excluded as none of the scans were determined to have any faces by either manual ratings or OpenCV.

Using the default confidence threshold of 0.5 (38) to determine whether or not an image contained a face, resulted in several noticeable disagreements with human raters on the presence of facial features (Figure 4). While this was mostly seen when only a single facial feature remained (shown as orange bars), there were still a number of full faces that fell below the threshold for detection (red bars), particularly among the mri_deface scans. In addition, there were a number of renders where a false face was detected (gray bars), where remnants of eye sockets, optic nerves, and other structures such as large tendons or blood vessels may have been mistaken for facial features by the detector, meaning that simply lowering the threshold will not solve these discrepancies between human and automated ratings.

**Figure 5:**
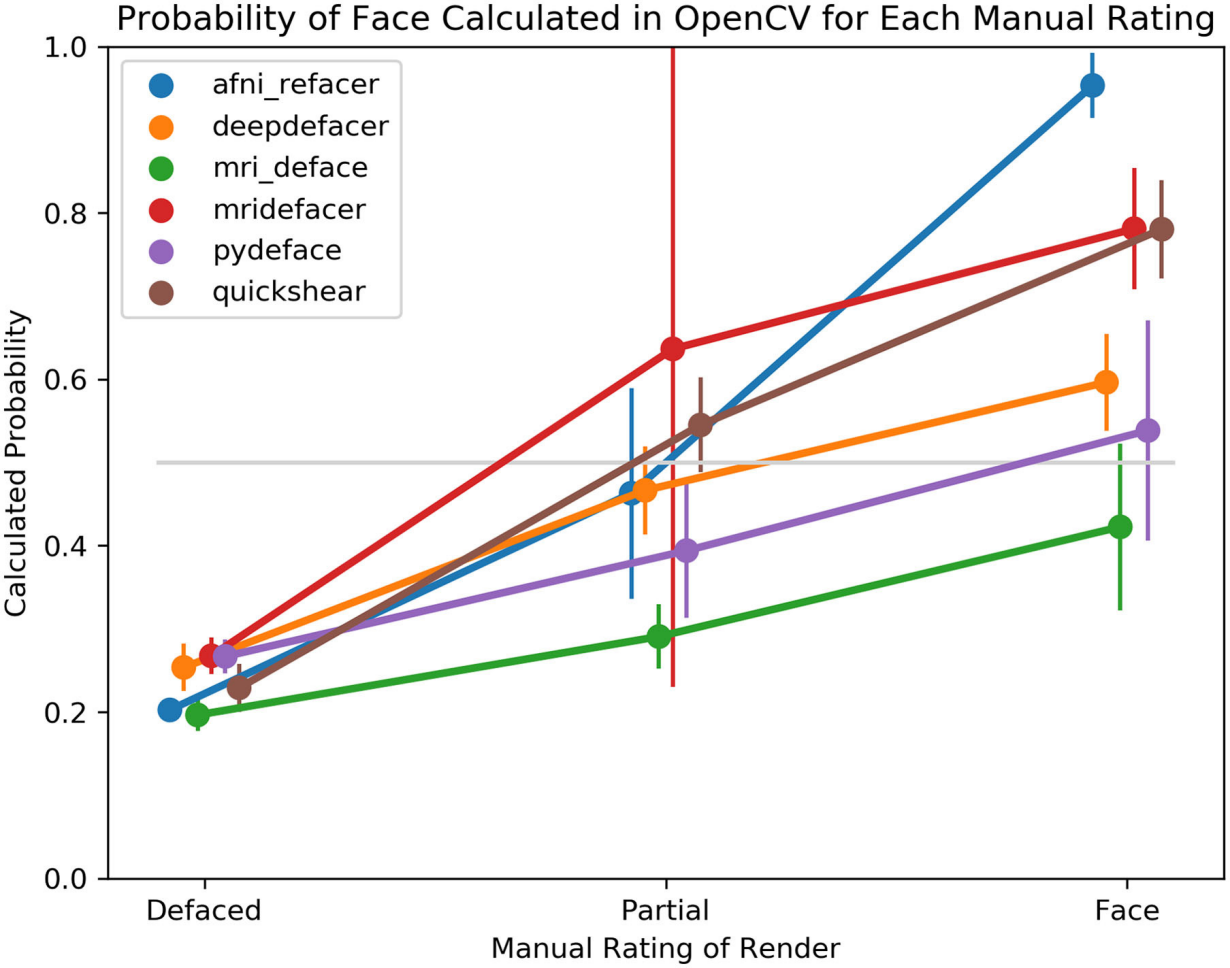
Average probability of a face within the 3D render as calculated by OpenCV, split based on manual rating consensus. Partial indicates scans where only one facial feature remained in the render, while Face indicates any scans where two or more features remained. FreeSurfer was excluded as all scans were rated as having been fully defaced. The gray line indicates the default threshold used by OpenCV to decide whether or not a face is present within the render.

Despite this, the overall trend for facial detection confidence rates matched what one would expect, with the average confidence for fully defaced scans well below the 0.5 threshold, while renders where human raters detected one visible feature, generated higher confidence levels, with even higher levels for those with two or more visible features (Figure 5). Besides the case of the fully defaced scans, these levels were not equal between defacers, ranging from an average confidence of 0.42 for mri_deface renders that still contained faces, to 0.95 for afni_refacer.

### Face Recognition

**Figure 6:**
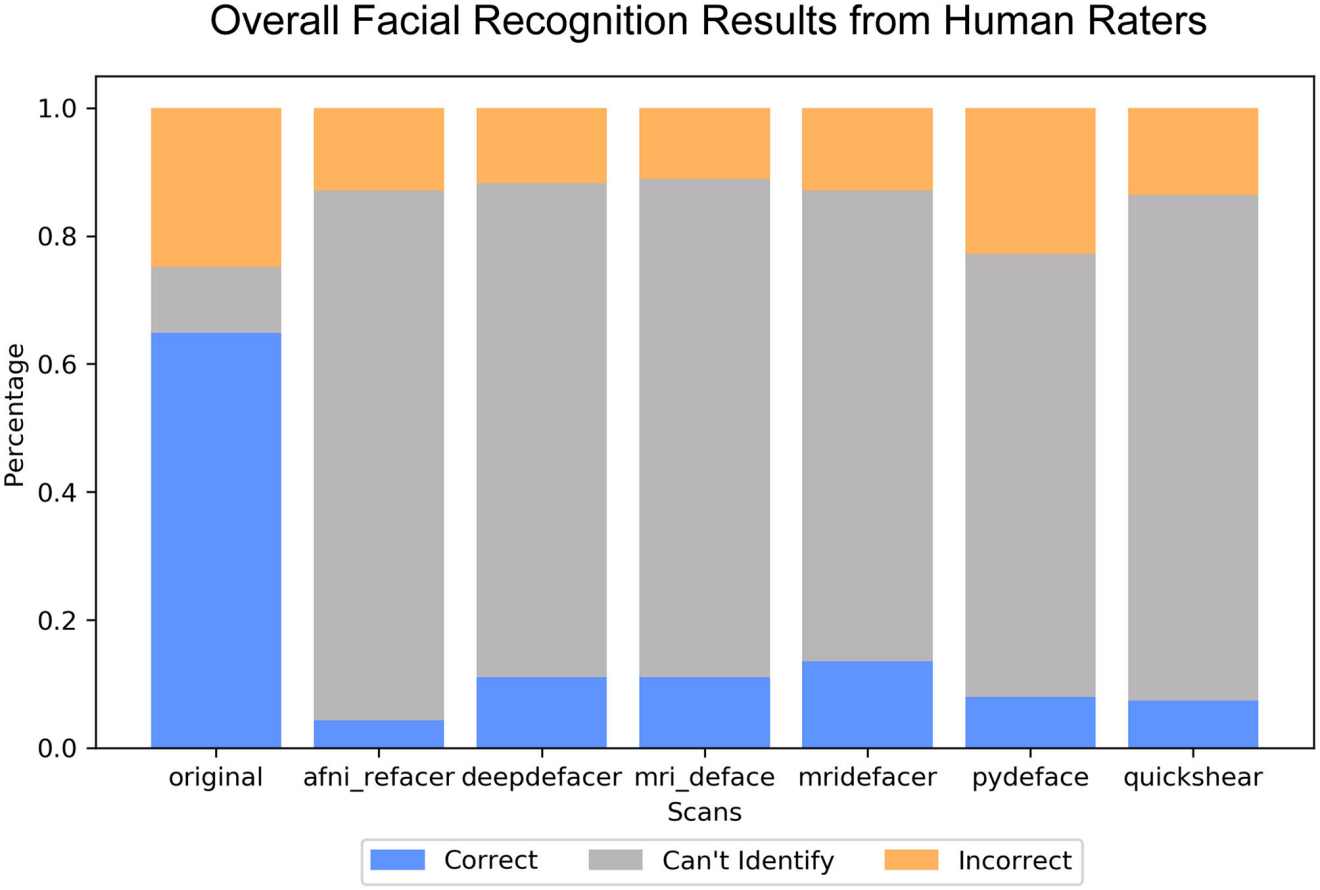
Bar plot indicating the percent distribution of facial recognition quiz results across the nine raters, for the renders of the defaced and original scans. Renders that were correctly matched to their corresponding photograph are considered “correct,” renders that were matched to the wrong photograph are considered “incorrect,” and those that the raters felt were not clear enough to attempt an identification are labeled “can't identify”.

Overall, defacing rendered the scans unrecognizable, with reviewers rating between 69.1 ± 34% (pydeface) to 82.7 ± 33% (afni_refacer) of renders to be completely unidentifiable (Figure 6), compared to the 10.3 ± 19% for the original scans. Correct identification was also very low, ranging from 4.3 ± 9% (afni_refacer) to 13.6 ± 11% (mridefacer), well below the 64.9 ± 18% for the original scans and was similar or lower than the rate at which the defaced renders were matched to the wrong photograph. This was particularly notable for pydeface, since although raters attempted to identify a much higher percentage of these renders than for the other defacers, only 26% of these attempted matches were actually correct.

The most commonly used features for identification, as reported by raters, were the nose (89%) and eyes (56%) for the renders of the original scans, while after defacing, eyebrows (56%) and skull shape (44%) were typically used in order to try to identify the participant within the renders.

### Influence on Preprocessing Pipelines

#### FreeSurfer Output

**Figure 7:**
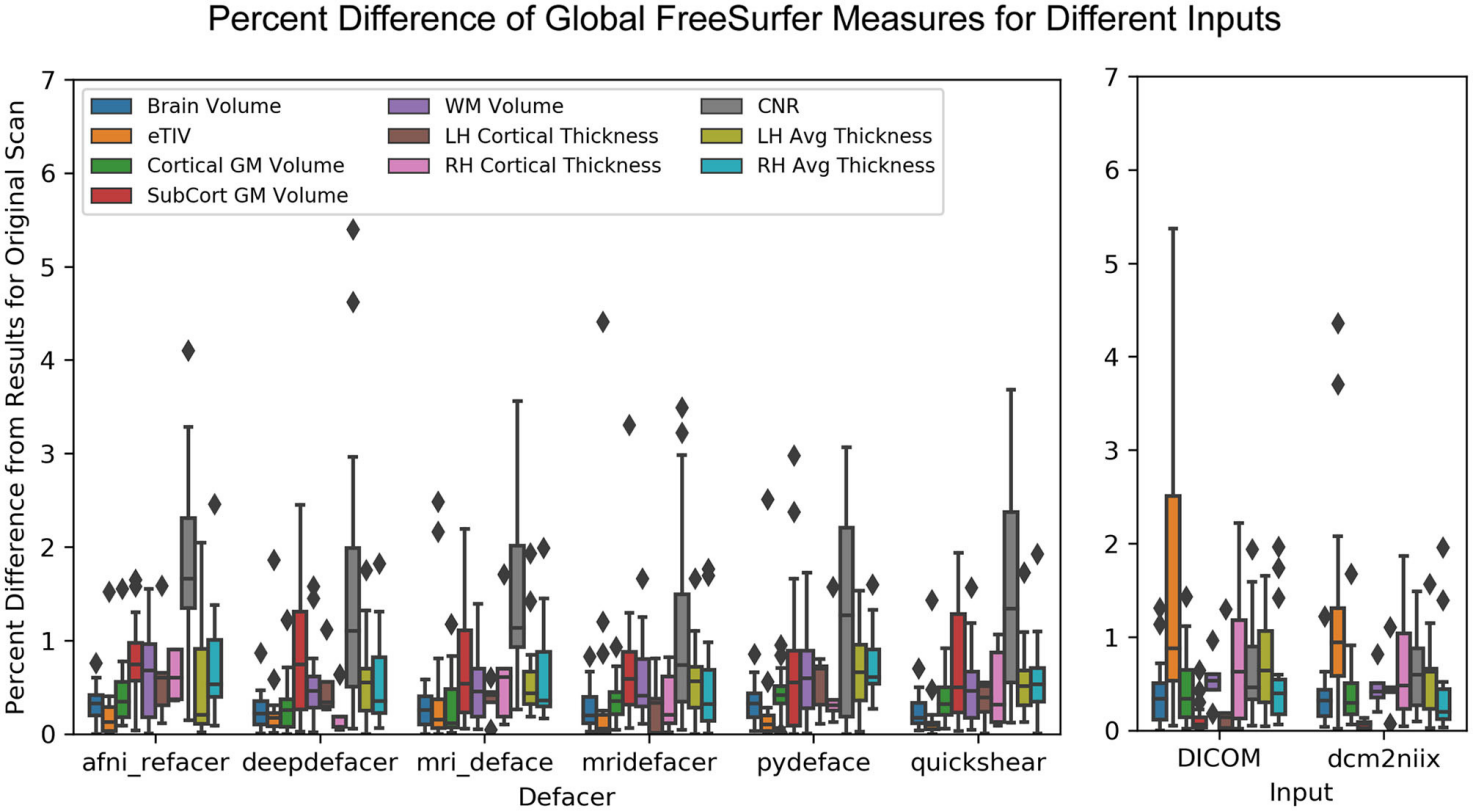
Boxplot showing the percent difference between each of the defaced images and the original pre-defaced scan (left) for several global measures generated from the FreeSurfer pipeline, including total brain volume, estimated total intracranial volume (eTIV), cortical and subcortical gray matter volumes, white matter volume, average left and right hemisphere cortical thickness and the contrast-to-noise ratio (CNR) between cortical gray and white matter. These are compared to the variations in the same measures (right) with FreeSurfer output initialized with the original scan in different file formats (raw DICOM and NIfTI files converted using a different method).

In most cases, while global measures were slightly different for the defaced images, these values only varied by ~1% from the original and were similar to the variation seen between the different versions of the pre-defaced scans (Figure 7). The exception to this was the gray-to-white matter CNR, which was frequently higher and as much as 5.4% different from the pre-defaced scan, as well as for one scan where mridefacer removed a small section of the back skull and upper dura, resulting in an estimated intracranial volume that was 4.4% lower than the original. DICOM and dcm2niix files saw similar differences, although it was the estimated intracranial volume that tended to vary more from the original scan, rather than CNR. A MANOVA revealed no significant difference [*F*_(64, 1,368)_ = 0.098934, *p* = 1] in global measures between any of the defaced or non-defaced scans.

**Figure 8:**
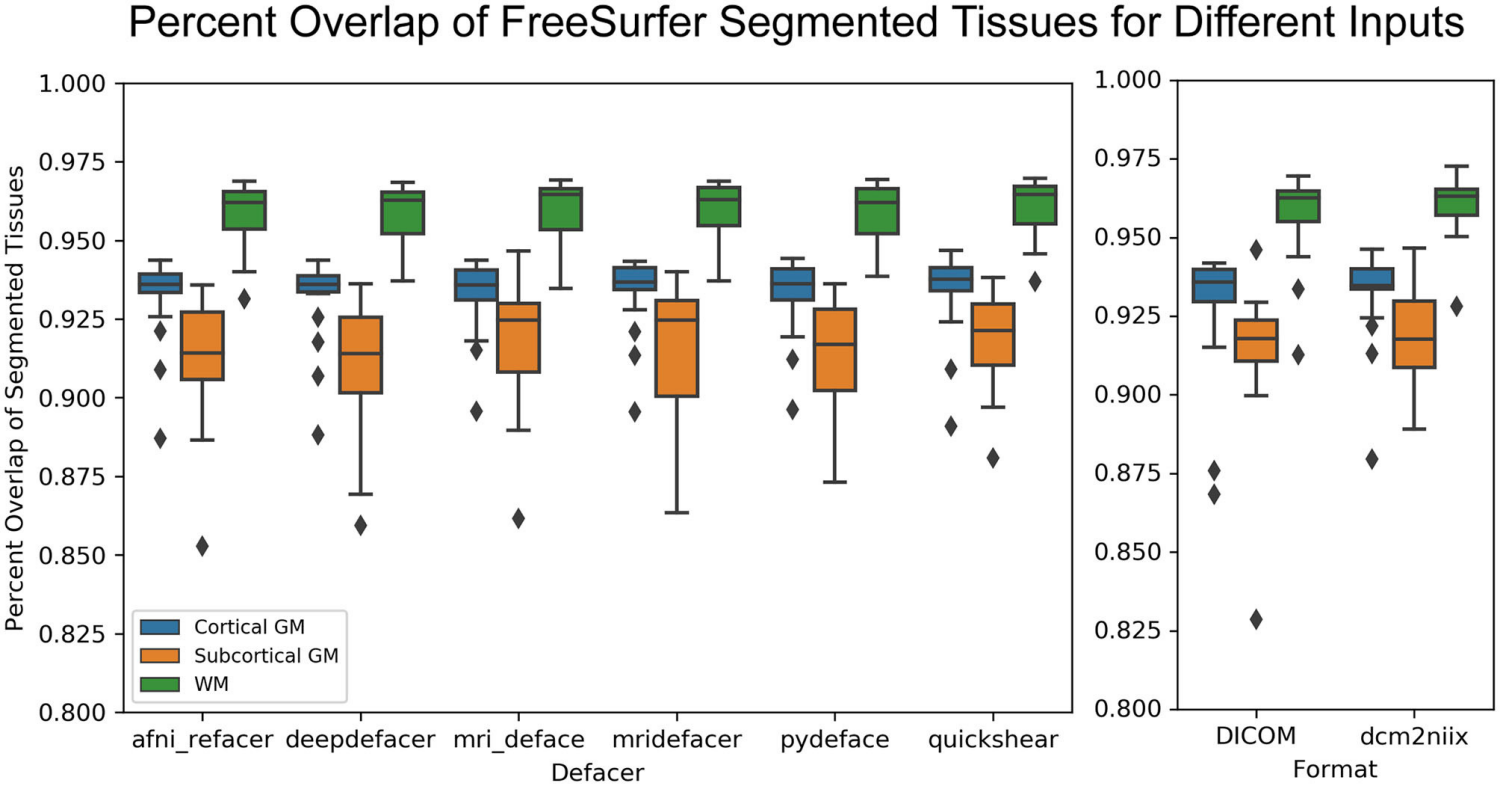
Boxplot showing the percent overlap of FreeSurfer segmented tissues for the defaced scans (left) and different file formats (raw DICOM and NIfTI files converted using alternate method, right), as defined by the area of the intersection with the original input scan, divided by the area of the union with the original, for that tissue.

While the defaced scans may have seen higher variability in CNR, the overlap of the actual segmented labels with the original scan was fairly high (>85% overlap for gray and white matter) and on par with that of the DICOM (>80% overlap) and dcm2niix (>85% overlap) file formats (Figure 8).

#### fMRI Preprocessing (OPPNI)

For the OPPNI pipeline, all general parameters—estimated head motion, optimal pipeline metrics, etc. were identical for all defaced and pre-defaced inputs. Although there were slight differences between the SPM files for each of the defaced and original scans, there were no voxel-wise significant differences after correcting for family-wise errors, and even raw *p*-values were only significant for a very small number of sparse voxels along the very edges of the brain.

#### Image Registration (FLIRT)

**Figure 9:**
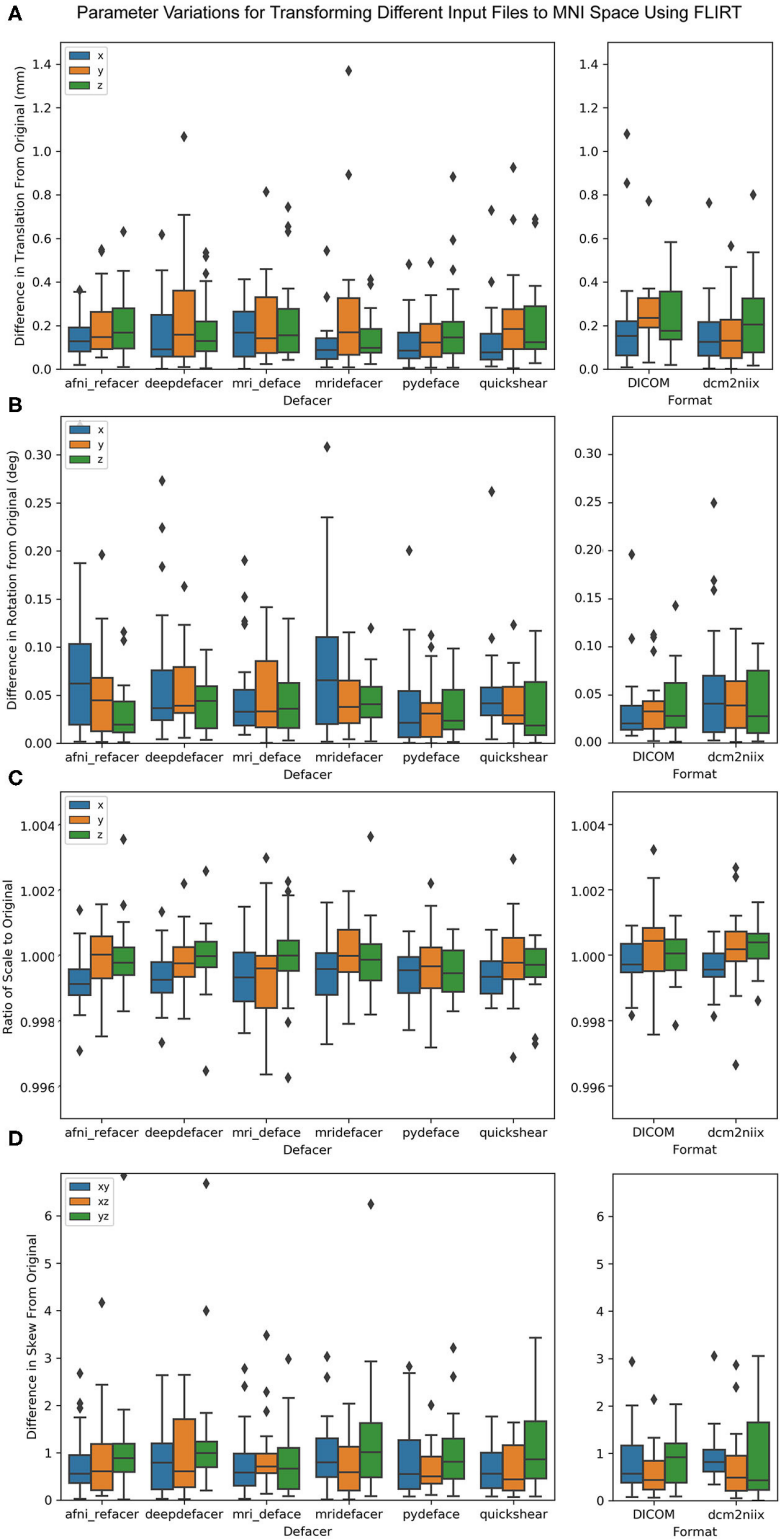
Boxplot of the difference between FLIRT parameters for the original scans and the defaced scans (left) and different file formats (right) when aligned to the MNI 152 brain template. Parameters have been split by translation in mm **(A)**, rotation converted to degrees **(B)**, scale **(C)**, and skew **(D)**.

When aligning the brain masks for the defaced scans to the MNI 152 template, all rotation parameters were less than a third of a degree different from the original brain masks, with no more than 1.5 mm difference in translation (Figure 9). There was also <0.5 percent difference in scale and 7.0 × 10^−3^ difference in skew, for all defacers. This was on par with the variation measured for DICOM and dcm2niix files. MANOVA results showed no significant difference [*F*_(96, 1,336)_ = 0.021, *p* = 1] between any of the defaced or non-defaced scans, for any of these parameters.

## Discussion

In this study, we sought to determine the best method for de-identifying MRI scans through a survey of existing publicly available algorithms. From our analyses, skull stripping seems to be the safest option for de-identifying structural T1s, both in terms of removing all identifiable features and for preserving brain tissue. However, for research studies where more than just the brain is required, afni_refacer and pydeface appear to be the most efficient defacers. The best choice for defacer seems to also depend on the data collected, with many of the defacers performing poorly with particular datasets; for example, afni_refacer's success rate was reduced with the youngest cohort (POND), while pydeface struggled with the oldest (ONDRI). These datasets were not the same across defacers, meaning this phenomenon is algorithm specific and not solely due to some inherent property of that dataset's scans that makes defacing difficult in general. Since there was a large degree of overlap between scanners among the three datasets tested here, this is also not a scanner specific issue, but a more complex interaction of participant age, diagnosis, defacing method, and other scan features. Practically, this is a useful trait, as scans that are unsuccessfully defaced by one algorithm, could still be defaced using another, instead of having to exclude them from shared datasets.

While there was an overall agreement between automated facial detection and human raters, there were some noticeable discrepancies, particularly for a few of the defacers (mri_deface, pydeface, deepdefacer) where even though the defacer failed to fully remove the participant's face, given OpenCV's low confidence that the render contained a face, it seems these algorithms still distorted the scan enough to confuse current software. Additionally, there were certain factors that appear more likely to fool OpenCV, either into detecting a face that is not there (traces of eye sockets, large blood vessels/tendons) or missing an existing face (noisy images, faces that have been squished or deformed by the head coil, goggles, etc.)

Visual facial recognition was quite low for defaced scans, with the majority not leaving enough features to even attempt matching with photographs. Of the scans where identification was attempted, only 25–51% of the matches were correct. While this is still higher than random chance, this does not indicate that these renders were highly recognizable, especially considering that raters were only dealing with scans from six volunteers. This rate was lowest for afni_refacer and pydeface, with these two also having among the least absolute number of correctly identified renders, aligning with our findings among the other three datasets that these two were the most successful at fully defacing scans. Additionally, these low identification rates were not due to the inherent difficulties of recognizing participants from their MRI renders, as the majority of the time, raters were able to correctly identify participants from the renders generated from the original, pre-defaced scans.

For the preprocessing pipelines tested, while there were slight differences between results using the original and the different defaced scans, the variations were very small and within the range of the differences between DICOM and NIfTI formats, or the two different NIfTI converters. The exception seemed to be for gray-to-white matter CNR for FreeSurfer intensity normalized data, which typically varied more from the original results for defaced scans than for the DICOM and dcm2niix files, possibly due to some of the defacing algorithms removing non-brain regions that were either hyperintense or suffered from signal dropout, leading to minor changes in the estimated bias field and overall intensity normalization. This issue is not exclusive to defaced scans, but also pertains to neuroimaging scans in general, where the presence or absence of extreme intensity values could introduce unwanted variances, supporting the use of pipelines which conduct intensity normalization based on a skull stripped image in order to increase consistency between scans.

While this is not conclusive evidence that defacing will never create discrepancies for subsequent analyses, for at least the majority of studies, any differences created by utilizing defaced scans will be negligible.

Other considerations, besides the accuracy of an algorithm at defacing scans and limiting the influence on the results of future analyses, include speed and additional software requirements (summarized in Table 6). The fastest methods were deepdefacer, mridefacer, and quickshear, taking only a couple of minutes per scan, although quickshear was only faster in terms of the actual defacing, as quickshear also requires a brain mask whose creation was not included in this estimate. Running pydeface and mri_deface took slightly longer, taking anywhere from 2 to 10 min per scan to finish, while the most successful software, @afni_refacer_run and FreeSurfer, could take roughly half an hour to complete.

**Table 6:** Completion time and prerequisites required for each tested algorithm.

**Software**	**Time to completion for one scan (min)**	**Prerequisites**
FreeSurfer	25–35	–
@afni_refacer_run	13–30	AFNI v20.0.02+, @afni_refacer_run v2.0+–older versions typically removed brain
deepdefacer	1–2	Python v2.7+ (numpy, nibabel, SimpleITK, TensorFlow, keras)
mri_deface	3–10	–
mridefacer	1–3	FSL, num-utils
pydeface	2–10	Python v2.7+ (numpy, nipype, nibabel), FSL
quickshear	~1 (does not include creation of brain mask)	Python v2.7+ (numpy, nibabel), brain mask

*Run times approximated when running on a 64-bit Intel® Core™ i7-4790 CPU @ 3.60 GHz processor using an Ubuntu 16.04 virtual machine with Windows 7 host*.

The software prerequisites for all of the tested algorithms are free, publicly available, and fairly straightforward to install, so in general, this should not present much of an issue when choosing which algorithm to go with. The one potential issue is that aside from deepdefacer and quickshear, all of the algorithms require either Linux or Mac OS, either for the application itself or for one of its prerequisites. Still, for Windows users, all of these applications will run on a Linux virtual machine, so again this should not be the main factor when deciding which defacing method to implement.

In conclusion, choice of the best defacer is dataset dependent, however, overall afni_refacer and pydeface have the highest success rates. Defacing scans has been shown to be an effective method in reducing participant recognizability, both in terms of automated facial detection and manual facial recognition, while resulting in only negligible changes to automated pre-processing pipeline results. Future work should explore the applicability and appropriateness of defacing software with other high-resolution structural images (e.g., T2-weighted), however, that is beyond the scope of this current manuscript.

## Data Availability Statement

The data analyzed in this study are subject to the following licenses/restrictions: Participants' data used in this study are currently stored in the Brain-CODE Neuroinformatics Platform (https://www.braincode.ca/) managed by the Ontario Brain Institute. Requests to access these datasets should be directed to the Ontario Brain Institute at info@braininstitute.ca.

## Ethics Statement

All recruitment sites adopted a standardized Participant Agreement with the OBI to enable the transfer of data in accordance with the Governance Policy of OBI as well as the local institutional and/or ethical policies. Written and informed parental consent was obtained for all participants under the age of 16. The patients/participants provided their written informed consent to participate in this study. Written informed consent was obtained from the individual(s) for the publication of any potentially identifiable images or data included in this article.

## Author Contributions

AT: creation of defaced images and 3D renders, review and rating of defaced scans, testing of preprocessing pipelines, statistical analysis, and drafting of manuscript. SA: review and rating of defaced scans, creation of facial recognition quiz and statistical analysis, and drafting of manuscript. MZ and MO'R: review and rating of defaced scans. JL and EA: POND data curation. CS, SS, and RB: ONDRI data curation. RL, BF, RM, DM, SK, SH, and GM: CANBIND data curation. SCS: development of initial research focus, guidance, and supervision for overall project. All authors have reviewed and approved the manuscript.

## Conflict of Interest

The authors declare that this study received funding from Lundbeck, Bristol-Myers Squibb, Pfizer, and Servier. The funders were not involved in the study design, collection, analysis, interpretation of data, the writing of this article, or the decision to submit it for publication. RM has received consulting and speaking honoraria from AbbVie, Allergan, Janssen, KYE, Lundbeck, Otsuka, and Sunovion, and research grants from CAN-BIND, CIHR, Janssen, Lallemand, Lundbeck, Nubiyota, OBI, and OMHF. RL has received honoraria or research funds from Allergan, Asia-Pacific Economic Cooperation, BC Leading Edge Foundation, CIHR, CANMAT, Canadian Psychiatric Association, Hansoh, Healthy Minds Canada, Janssen, Lundbeck, Lundbeck Institute, MITACS, Myriad Neuroscience, Ontario Brain Institute, Otsuka, Pfizer, St. Jude Medical, University Health Network Foundation, and VGH-UBCH Foundation. SCS is the Chief Scientific Officer of ADMdx, Inc., which receives NIH funding, and he currently has research grants from Brain Canada, Canada Foundation for Innovation (CFI), Canadian Institutes of Health Research (CIHR), and the Ontario Brain Institute in Canada. BF has received a research grant from Pfizer. SK has received research funding or honoraria from Abbott, Alkermes, Allergan, Bristol-Myers Squibb, Brain Canada, Canadian Institutes for Health Research (CIHR), Janssen, Lundbeck, Lundbeck Institute, Ontario Brain Institute (OBI), Ontario Research Fund (ORF), Otsuka, Pfizer, Servier, Sunovion, and Xian-Janssen. EA has served as a consultant to Roche, has received grant funding from Sanofi Canada and SynapDx, has received royalties from APPI and Springer, and received kind support from AMO Pharmaceuticals, honoraria from Wiley, and honorarium from Simons Foundations. GM has received consultancy/speaker fees from Lundbeck, Pfizer, Johnson & Johnson and Janssen. The remaining authors declare that the research was conducted in the absence of any commercial or financial relationships that could be construed as a potential conflict of interest.
