# Supplementary material for: Multisite Comparison of MRI Defacing Software Across Multiple Cohorts
Source: Front Psychiatry. 2021 Feb 24;12:617997. doi: 10.3389/fpsyt.2021.617997 (PMC7943842; doi:10.3389/fpsyt.2021.617997)
Supplement: Supplementary file 1 [file Data_Sheet_1.docx]

Supplementary Material 1


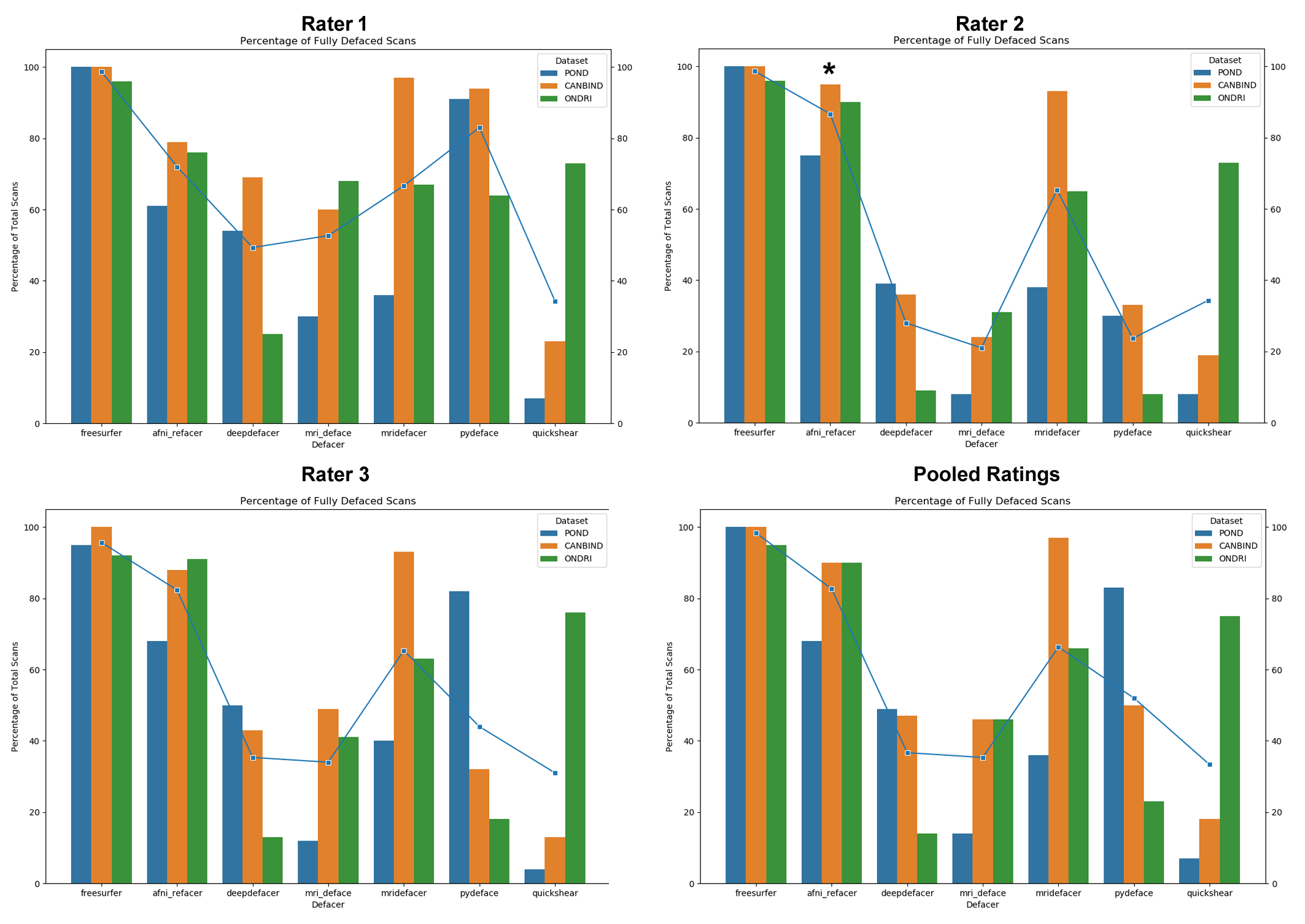


**Supplementary Figure 1.** Percentage of scans rated as having no brain removed and no facial features remaining, split by dataset and defacing algorithm. Markers indicate the average percentage for each algorithm. Pooled ratings indicate the percentage of scans that passed based on rater consensus for each scan. *Disclaimer: afni_refacer_run ratings had to be redone due to a major software update after initial data collection. Due to the unavailability of the original Rater 2, these ratings were completed by a different person.
