# Supplementary material for: Multisite Comparison of MRI Defacing Software Across Multiple Cohorts
Source: Front Psychiatry. 2021 Feb 24;12:617997. doi: 10.3389/fpsyt.2021.617997 (PMC7943842; doi:10.3389/fpsyt.2021.617997)
Supplement: Supplementary file 2 [file Data_Sheet_2.docx]

Supplementary Material 2

**Supplementary Table 1.** Inter-rater reliability for manual ratings of each dataset and algorithm, as measured using percent agreement and free-marginal kappa, using a stricter threshold of no features remaining to be considered a pass

|  | Percent Agreement | | | Free Marginal Kappa | | |
| --- | --- | --- | --- | --- | --- | --- |
| Defacer | POND | CANBIND | ONDRI | POND | CANBIND | ONDRI |
| FreeSurfer | 96.7 | 100 | 94.0 | 0.933 | 1.00 | 0.880 |
| afni_refacer | 92.0 | 84.7 | 88.7 | 0.840 | 0.920 | 0.773 |
| deepdefacer | 81.3 | 68.0 | 87.3 | 0.627 | 0.360 | 0.747 |
| mri_deface | 80.0 | 68.7 | 70.7 | 0.600 | 0.373 | 0.413 |
| mridefacer | 92.0 | 94.7 | 94.0 | 0.840 | 0.893 | 0.880 |
| pydeface | 56.0 | 47.3 | 59.3 | 0.120 | -0.053 | 0.187 |
| quickshear | 96.0 | 90.0 | 95.3 | 0.920 | 0.800 | 0.907 |
