# Supplementary material for: Multisite Comparison of MRI Defacing Software Across Multiple Cohorts
Source: Front Psychiatry. 2021 Feb 24;12:617997. doi: 10.3389/fpsyt.2021.617997 (PMC7943842; doi:10.3389/fpsyt.2021.617997)
Supplement: Supplementary file 3 [file Data_Sheet_3.docx]

Supplementary Material 3

**Supplementary Table 2:** Example 3D renders of successful defaced and failed scans including the listed features that the defacer missed. Defacing shown on a T1 template scan to preserve participant privacy

| Defacer | Example - Success | Example - Fail | Errors in Fail Example |
| --- | --- | --- | --- |
| afni_refacer_run | 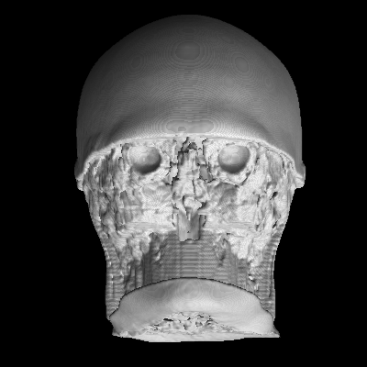 | 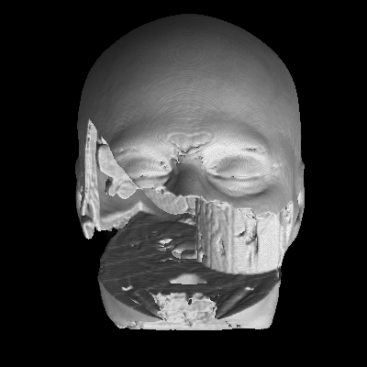 | Eyes and part of nose remaining; section of brain has also been removed |
| deepdefacer | 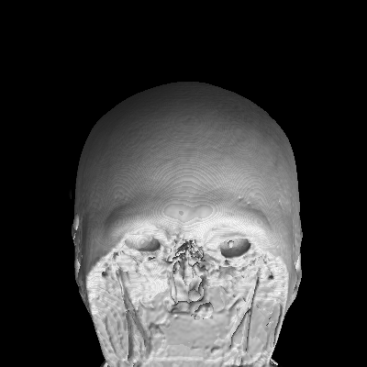 | 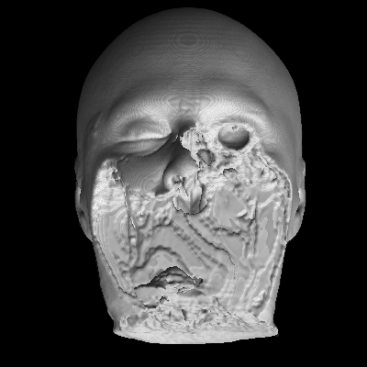 | Right eye and part of nose remaining |
| mri_deface | 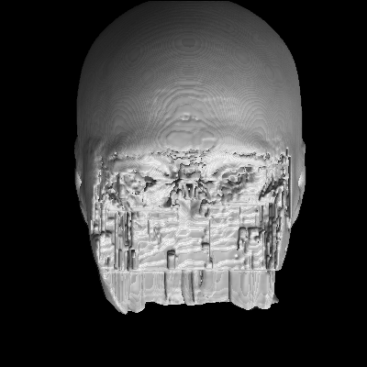 | 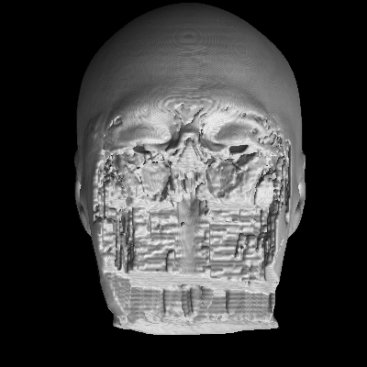 | Eyes remaining |
| mridefacer | 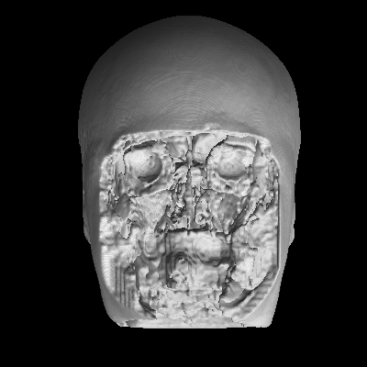 | 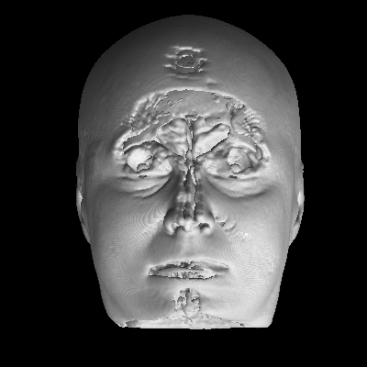 | Mouth, most of nose and bottom portion of eyes remaining |
| pydeface | 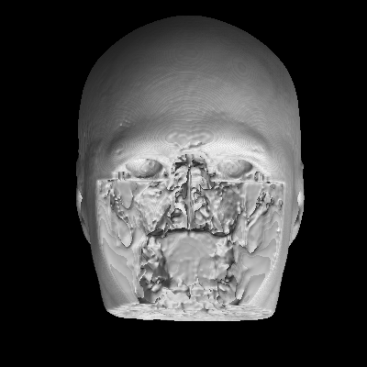 | 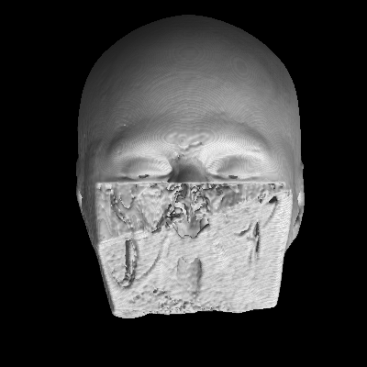 | Eyes and top of nose remaining |
| quickshear | 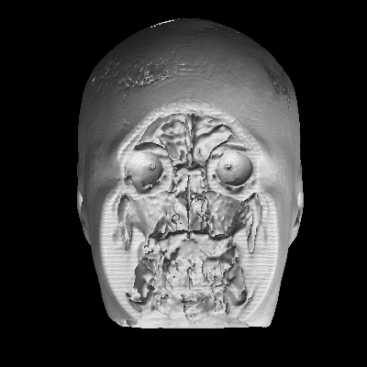 | 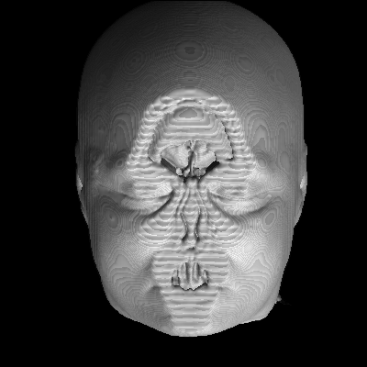 | Eyes and corners of mouth remaining |
